# Activation of transient receptor potential vanilloid 3 channel (TRPV3) aggravated pathological cardiac hypertrophy via calcineurin/NFATc3 pathway in rats

**DOI:** 10.1111/jcmm.13880

**Published:** 2018-10-09

**Authors:** Qianhui Zhang, Hanping Qi, Yonggang Cao, Pilong Shi, Chao Song, Lina Ba, Yunping Chen, Jingquan Gao, Shuzhi Li, Baiyan Li, Hongli Sun

**Affiliations:** ^1^ Department of Pharmacology Harbin Medical University‐Daqing Daqing Heilongjiang China; ^2^ Department of Nursing Harbin Medical University‐Daqing Daqing Heilongjiang China; ^3^ Department of Pharmacology Harbin Medical University Harbin Heilongjiang China

**Keywords:** Ca^2+^, Calcineurin, Cardiac Hypertrophy, NFATc3, Transient receptor potential vanilloid 3

## Abstract

Cardiac hypertrophy is a compensatory response to mechanical stimuli and neurohormonal factors, ultimately progresses to heart failure. The proteins of some transient receptor potential (TRP) channels, Ca^2+^‐permeable nonselective cation channel, are highly expressed in cardiomyocytes, and associated with the occurrence of cardiac hypertrophy. Transient receptor potential vanilloid 3 (TRPV3) is a member of TRP, however, the functional role of TRPV3 in cardiac hypertrophy remains unclear. TRPV3 was elevated in pathological cardiac hypertrophy, but not in swimming exercise‐induced physiological cardiac hypertrophy in rats. TRPV3 expression was also increased in Ang II–induced cardiomyocyte hypertrophy in vitro, which was remarkably increased by carvacrol (a nonselective TRPV channel agonist), and reduced by ruthenium red (a nonselective TRPV channel antagonist). Interestingly, we found that activated TRPV3 in Ang II–induced cardiomyocyte hypertrophy was accompanied with increasing intracellular calcium concentration, promoting calcineurin, and phosphorylated CaMKII protein expression, and enhancing NFATc3 nuclear translocation. However, blocking or knockdown of TRPV3 could inhibit the expressions of calcineurin, phosphorylated CaMKII and NFATc3 protein by Western blot. In conclusion, the activation of TRPV3 aggravated pathological cardiac hypertrophy through calcineurin/NFATc3 signalling pathway and correlated with the protein expression levels of calcineurin, phosphorylated CaMKII and NFATc3, revealing that TRPV3 might be a potential therapeutic target for cardiac hypertrophy.

## 1 INTRODUCTION

Cardiac hypertrophy is a compensatory response to mechanical stimuli and neurohormonal factors. The terminal of cardiac hypertrophy often ends with heart failure or sudden death, leading to a high mortality in the whole world.^1^ In response to the workload of the heart, consequently, left ventricular (LV) is worsening, cell sizes are increasing and foetal cardiac genes are reprogramming.^2^ Pathological and physiological hypertrophy can be classified by different initiating stimulus, following several signal pathways. To date, accumulating studies showed that the Gαq pathway activated pathological hypertrophy including Gαq/PLC/Ca^2+^, PKC pathway, gp130/JAK/STAT signal pathway, and the IGF1‐PI3K pathway activated physiological hypertrophy.^3^ Although several signal‐transduction pathways contributed to the hypertrophic process have been studied in recent years, it is still not fully to clear underlying molecular mechanisms for cardiac hypertrophy.

The transient receptor potential (TRP) superfamily of cation channels contains six subfamilies, among which most of them could be permeable to Ca^2+^.^4^ In TRP subfamily, such as TRPC channels are developed in cardiac hypertrophy, especially TRPC3 is increased in several animal models of cardiac hypertrophy and promotes cardiomyocyte hypertrophy.^5^ TRPV2, TRPM7, and TRPP2 are also associated with cardiomyopathy and cardiac fibrosis.^6^ As a member of the TRP family, transient receptor potential vanilloid 3 (TRPV3) channel has been reported that it is expressed in skin keratinocytes, oral, dorsal root ganglion, trigeminal ganglion, spinal cord, brain, and nasal epithelia.^7^ In our experiment, we confirmed that the existence of TRPV3 in heart and also revealed that TRPV3 was activated in hypertrophic myocardium of rat heart induced by pressure overload. Therefore, we speculated that the TRPV3 could be directly or indirectly involved in cardiac hypertrophy. However, the effects and mechanisms of TRPV3 on cardiac hypertrophy need to be further explored.

Calcineurin/nuclear factor of activated T cell (NFAT) pathway plays a key role in electrical remodelling in pathological cardiac hypertrophy. Calcineurin is a cytoplasmic Ca^2+^/calmodulin‐actived protein phosphatase that modulates hypertrophy response. Increased Ca^2+^ levels activate calcineurin, which binds to transcription factors of the nuclear factor of activated T cell c3 (NFATc3). Upon binding, it dephosphorylates NFATc3 to the nucleus and activates pro‐hypertrophic genes expression.^8^

In view of TRPV3 being a nonselective cation channel,^7^ whether it is permeable to Ca^2+^, and contributes to cardiac hypertrophy via calcineurin/NFAT signaling pathway, thus, we aimed to investigate the effects of TRPV3 in pressure overload‐induced cardiac hypertrophy, and to clarify the relationship between TRPV3 activation and calcineurin/NFAT pathway.

## 2 MATERIALS AND METHODS

### 2.1 Ethics statement

Male adult Wistar rats, weighing 225‐250 g, were provided by the Animal Research Center of Harbin Medical University. All animal protocols were performed with the approval of the Experimental Animal Ethic Committee of Harbin Medical University, China. The study was carried out in accordance with the Guide for the Care and Use of Laboratory Animals published by the US National Institutes of Health (NIH Publication, 8th Edition, 2011).

### 2.2 Swimming exercise‐induced physiological cardiac hypertrophy

Physiological hypertrophy rats were performed over 4 weeks in tracking system containing warmed water (30‐32°C), as reported.^9^ Briefly, animals were made to swim in this system for 90 min/d, 6 d/wk. At the beginning, animals were trained to swim for adaption 1 week. Adaption training was set at 15 minutes on day 1, gradually increased by 15 minutes every other day until 90 minutes, 6 days were reached. Then animals were subjected to swim for 4 weeks. Twenty‐four hours after the last exercise session, in the condition of anesthetization with pentobarbital sodium (40 mg/kg, i.p.), the rats were sacrificed by cervical dislocation, and then the hearts were removed and immediately weighed. Tibial length was also measured by vernier caliper. Then the heart weight/tibial length ratio (HW/TL; mg/mm) was made. The left ventricle and right ventricle were separated and the left ventricle tissue was taken and washed by normal saline, then was stored at −80°C for subsequent experiment.

### 2.3 Pressure overload‐induced pathological cardiac hypertrophy

The pressure‐overload hypertrophy was subjected to abdominal aortic constriction (AAC) as previously described.^10^ Briefly, rats were anesthetized with pentobarbital sodium (40 mg/kg, i.p.). To expose abdominal aorta and renal arteries, a midline incision of 1–2 cm was made in abdomen. The surgical suture was passed beneath the abdominal aorta and tied along with a blunted 5 gauge needle. To ensure consistent constricted, the needle was withdrawn to leave the abdominal aorta immediately. The sham‐operated group underwent the same surgical procedures but without ligation. Rats were kept under observation for 2 and 4 weeks. Two and 4 weeks after surgery, the hearts were harvested and weighed to calculate left ventricular weight/body weight ratio (LVW/BW; mg/g), the heart weight/body weight ratio (HW/BW; mg/g) and the heart weight/tibial length ratio (HW/TL; mg/mm), then were stored at −80°C for subsequent experiment.

### 2.4 Hemodynamics

Left ventricular function was continuously recorded with BL‐420N organism function experiment system (Cheng Du Tai Meng, China). 0.9% NaCl with 5 IU/mL heparin composed a catheter which was inserted into the right carotid artery and then advanced into the left ventricle. The system dictated were left ventricular systolic pressure (LVSP), left ventricular end‐diastolic pressure (LVEDP), and maximum rate of left ventricular pressure rise and fall (+dp/dt_max_ and −dp/dt_max_).

### 2.5 Immunohistochemistry

Sham rats and AAC rats in two or four weeks after ligation were prepared. Following anaesthesia, the hearts were taken out and immediately placed in 4% paraformaldehyde at 4°C overnight. The left ventricle was embedded in paraffin, cut into 5 μm sections. Some sections were stained with haematoxylin and eosin (H&E). For immunohistochemistry, sections were dewaxed and dehydrated in 100%, 95%, 90%, 85%, 80%, and 75% ethanol each 2 minutes. All slices were incubated 3% hydrogen peroxide for 10 minutes and heated for 10 minutes for antigen retrieval. After sections were allowed to cool to room temperature and added to the goat serum sealing fluid at 37°C for 30 minutes. Subsequently, the sections were incubated with an anti‐TRPV3 antibody (1:200; Alomone labs, Jerusalem, Israel) overnight at 4°C. Then the sections were incubated to secondary antibody for 1 hour. The sections were visualized with a diaminobenzidine (DAB) and counterstained with haematoxylin.

### 2.6 Primary culture of neonatal rat cardiomyocytes

Neonatal rat ventricular myocytes were isolated as previously described.^11^ Three‐day‐old neonatal Wistar rats were scarified by cervical dislocation, and then dipped into 75% ethanol solution for 1‐2 minutes. After that, the hearts were rapidly cleaned, moved out, and cut into pieces. Then, all the tissues which were inhaled in 15 mL centrifuge tubes were treated with 0.25% trypsin solution (provided by Beyotime) for 5 minutes, the digested supernatant was collected with DMEM (Dulbecco's modified eagle's medium) supplemented with 10% foetal bovine serum and stored at 4°C. Until all the tissues were disappeared, the digested supernatant was collected in the end. All the cells in supernatants were filtered with a 200 mesh sieve, centrifuged at 425 ***g*** 10 minutes and replaced at culture medium. Cardiomyocytes and nonmyocytes were separated at 1.5 hours. Then the cardiomyocytes were incubated at new culture media 48 hours. Neonatal rat cardiomyocytes were randomly divided into six groups, respectively: (a) control group; (b) model group: cells were incubated with Ang II (100 nmol L^−1^; Sigma, St. Louis, MO, USA) for 48 hours; (c) model + carvacrol group: cells were given 60 μmol L^−1^ carvacrol (Car, a nonselective TRPV channel agonist, Sigma) for 4 hours after treatment with 100 nmol L^−1^ Ang II for 48 hours; (d) model + ruthenium red + carvacrol group: 20 μmol L^−1^ ruthenium red (RuR, a nonselective TRPV channel antagonist; Sigma) was administered for 2 hours before 60 μmol L^−1^ carvacrol, following 48 hours treatment with 100 nmol L^−1^ Ang II; (e) control + carvacrol group: cells were given 60 μmol L^−1^ carvacrol for 4 hours; (f) model + ruthenium red group: 20 μmol L^−1^ ruthenium red was added for 2 hours after 48 hours treatment with 100 nmol L^−1^ Ang II.

### 2.7 Ca^2+^ fluorescence measurement

Fluorescence measurements in cardiomyocytes have been described previously.^12^ Cultured cardiomyocytes were incubated with Fluo‐3/AM (10 μmol L^−1^) working solution containing 0.03% Pluronic F‐127 at 37°C in the dark for 45 minutes. Then the cardiomyocytes were washed twice with Tyrode solution to remove the extracellular Fluo‐3/AM. Fluorescent intensity in intracellular calcium ions was recorded by laser scanning confocal microscope (Olympus, Tokyo, Japan) with 488 nm for excitation and 530 nm for emission. Fluorescent intensity was measured in 10 randomly cells to calculate the average FI.

### 2.8 Measurement of cell surface area

Cultured cardiomyocytes were washed in PBS, fixed for 10 minutes, in 4% paraformaldehyde for 15 minutes and then permeabilized by 0.2% Triton X‐100 for 20 minutes and blocked by 1% bovine serum albumin at 37°C for 40 minutes. After that, the cells were followed by anti‐sarcomeric actin antibody (1:200; Santa Cruz Biotechnology, Santa Cruz, CA, USA) at 4°C overnight and subsequently with a Cy3‐conjugated goat anti‐mouse antibody (1:1000; Sigma) for 2 hours at room temperature and DAPI for 15 minutes. Immunofluorescence was analysed under a fluorescence microscope (Nikon, 80i, Tokyo, Japan).

### 2.9 Western blotting

The procedure was adapted from our previous studies.^13^ Total proteins from the tissues and cells were extracted with lysis buffer including 1% protease inhibitor solution. Briefly, the protein concentrations were determined by BCA protein assay kit. Protein samples (100 mg) were separated by 10% SDS‐PAGE and transferred to nitrocellulose membranes and then blocked with 5% nonfat milk. The membranes were probed overnight at 4°C with the following antibodies: TRPV3 (1:200; Alomone labs, Jerusalem, Israel), p‐CaMKII and CAMKII (1:1000; Cell Signaling Technology, Boston, MA, USA), calcineurin (1:1000; Cell Signaling Technology), β‐MHC (1:3000; Sigma), NFATc3 (1:100; Santa Cruz Biotechnology, Santa Cruz, CA, USA), BNP (1:100; Santa Cruz Biotechnology), Hsp70 (1:300; Boster Inc., Wuhan, China), β‐actin (1:2000; Zhong Shan‐Golden Bridge Biological Technology, Beijing, China), GAPDH (1:2000; Zhong Shan‐Golden Bridge Biological Technology). β‐actin or GAPDH was as a loading control. After washing three times, members were incubated with a horseradish peroxidase‐conjugated secondary anti‐rabbit/mouse/goat IgG for 2 hours at room temperature. Proteins were visualized using ECL (Thermo Fisher Scientific, Inc., Waltham, MA, USA), and collected by using the Bio Imaging Systems (UVP Inc., Upland, CA, USA).

### 2.10 Immunocytochemistry

Neonatal rat ventricular myocytes were cultured on coverslips, which were covered in 6‐well culture plates. After 48 hours treatment, cells were placed in 4% paraformaldehyde and then permeabilized by 0.2% Triton X‐100 for 10 minutes and blocked by 1% bovine serum albumin at 37°C for 40 minutes. After that, cells were incubated with anti‐TRPV3 primary antibody (rabbit, 1:100) at 4°C overnight. After washed three times with PBS, the cells were subsequently incubated with a FITC‐conjugated goat anti‐mouse antibody (1:100) for 2 hours at room temperature and DAPI for 15 minutes. Immunofluorescence was analysed under a fluorescence microscope (Nikon, 80i, Tokyo, Japan).

### 2.11 Cell transfection

To block the expression of TRPV3 protein, neonatal rat ventricular myocytes were transfected with the X‐treme GENE siRNA transfection reagent (Roche, Penzberg, Germany). NC siRNA (GenePharma Co., Ltd., Shanghai, China), non‐targeted control siRNA, was used as the negative control. As below, the sense sequence of siRNA against TRPV3 was listed: 5′‐ACCUGCCUGAUGAAAGCUUTT‐3′ and 5′‐AAGCUUUCAUAGGCAGGUTT‐3′. Cardiomyocytes were transfected with siRNA TRPV3 for 24 hours, and then the transfection reagents were removed and cells were cultured constantly in DMEM with 10% FBS. Cells were divided into four groups: (a) control group; (b) model group: cells were incubated with Ang II (100 nmol L^−1^) for 48 hours; (c) siRNA TRPV3 + Ang II: after 24 hours siRNA TRPV3 transfection, cells were treated with Ang II (100 nmol L^−1^) for 48 hours; (d) NC siRNA + Ang II: cells were transfected with NC siRNA for 24 hours, then were incubated with Ang II (100 nmol L^−1^) for 48 hours.

### 2.12 Statistical analysis

Data were presented as means ± SD. Statistical analysis was performed with one‐way ANOVA followed by Tukey's test where appropriate. Student's *t* test was used for comparisons between two groups. *P *< 0.05 was considered statistically significant.

## 3 RESULTS

### 3.1 TRPV3 expression was increased in pathological cardiac hypertrophy, not in physiological cardiac hypertrophy

**Figure 1:**
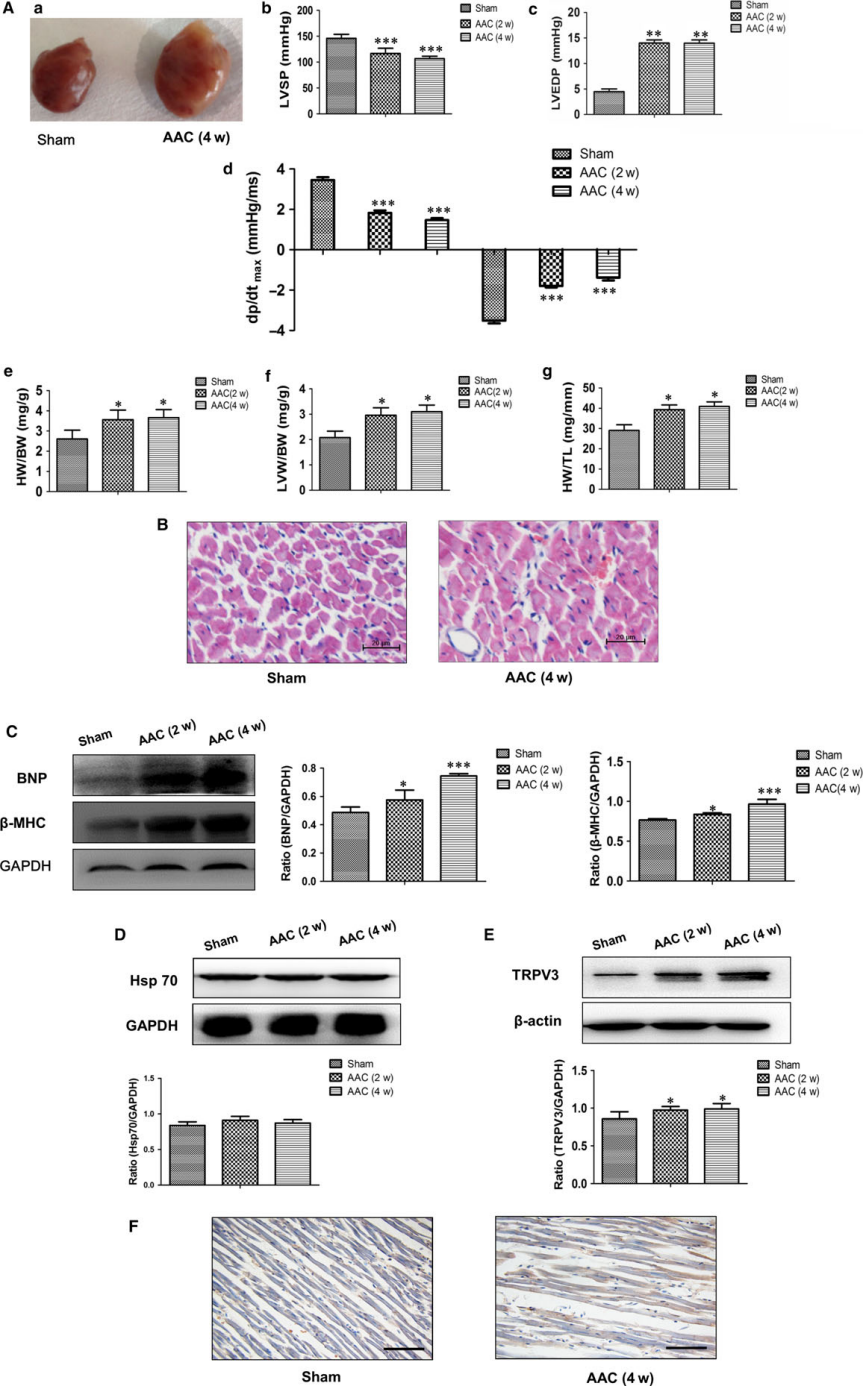
Transient receptor potential vanilloid 3 (TRPV3) expression was increased in pathological cardiac hypertrophy induced by abdominal aortic constriction (AAC) in vivo. A‐a, The whole size of the heart from AAC 4 w was larger than that in sham group. A‐b, c and d, LVSP, LVEDP, +dp/dt_max_ and −dp/dt_max_ were measured throughout the experiment. LVSP, LVEDP and ±dp/dt_max_ represent left ventricular systolic pressure, left ventricular end‐diastolic pressure, and the maximal rates of increase and decrease in LV pressure, respectively. A‐e, The ratio of heart weight to body weight (HW/BW). A‐f, The ratio of left ventricle weight to body weight (LVW/BW). A‐g, The ratio of heart weight to tibial length (HW/TL). B, HE staining of sections from sham group and AAC 4 w group to detect hypertrophy (×200). C, The expressions of BNP and β‐MHC after AAC 2 and 4 weeks. D, Hsp70 protein was not changed in AAC group. E, TRPV3 expression in rat hypertrophy myocardium after AAC 2 and 4 weeks. F, Immunohistochemical staining of TRPV3 was examined in hypertrophy myocardium (×200). Brown stain represented positive signal. Immunoreactive TRPV3 was mainly expressed in cell membrane. *P < 0.05, **P < 0.01, ***P < 0.001 vs sham group. Data were presented by mean ± SD (n = 6)

Pressure‐overload hypertrophy for 2 and 4 weeks was induced by AAC in rats. Strikingly, the whole sizes of the hearts from AAC for 4 w rats were markedly larger than those from sham group (Figure 1A‐a). LVSP and ±dp/dt_max_ in the AAC‐2 w and AAC‐4 w groups both decreased (Figure 1A‐b, A‐d), whereas, LVEDP increased significantly compared with sham group (Figure 1A‐c, *P *< 0.01). Cardiac hypertrophy was further confirmed by HW/BW, LVW/BW, HW/TL and the morphology (Figure 1A‐e, f, g). As shown in Figure 1A, HW/BW, LVW/BW, and HW/TL were significantly increased in the AAC‐2 w (*P *< 0.05) and AAC‐4 w groups (*P *< 0.05) compared with sham group. Observed with light microscope, H&E stain showed that the cardiomyocytes were hypertrophic and arranged irregularly in AAC groups (Figure 1B). Pathological cardiac hypertrophy is marked by the induction of protein normally expressed during foetal development, such as brain natriuretic peptide protein (BNP) and β‐myosin heavy chain (β‐MHC). Therefore, the expressions of BNP and β‐MHC were detected by Western blot analysis. The results showed that dramatic increases in BNP and β‐MHC of the hearts rats as early as 2 weeks in the AAC surgery were observed, and more highly enhanced at 4 weeks after AAC surgery (*P *< 0.001) (Figure 1C). The above results indicated that pathological cardiac hypertrophy was established successfully. We next measured TRPV3 expression in rat hypertrophy myocardium using Western blot. Our results revealed that TRPV3 was significantly increased in myocardium of rats in the AAC‐2 w and AAC‐4 w groups compared with sham group (*P *< 0.05) (Figure 1E). We also located TRPV3 in the heart using immunohistochemistry on tissue sections. The expression of TRPV3 was elevated in AAC group, which was mainly located on cell membrane (Figure 1F). Taken together, TRPV3 expression was increased in pathological hypertrophy.

**Figure 2:**
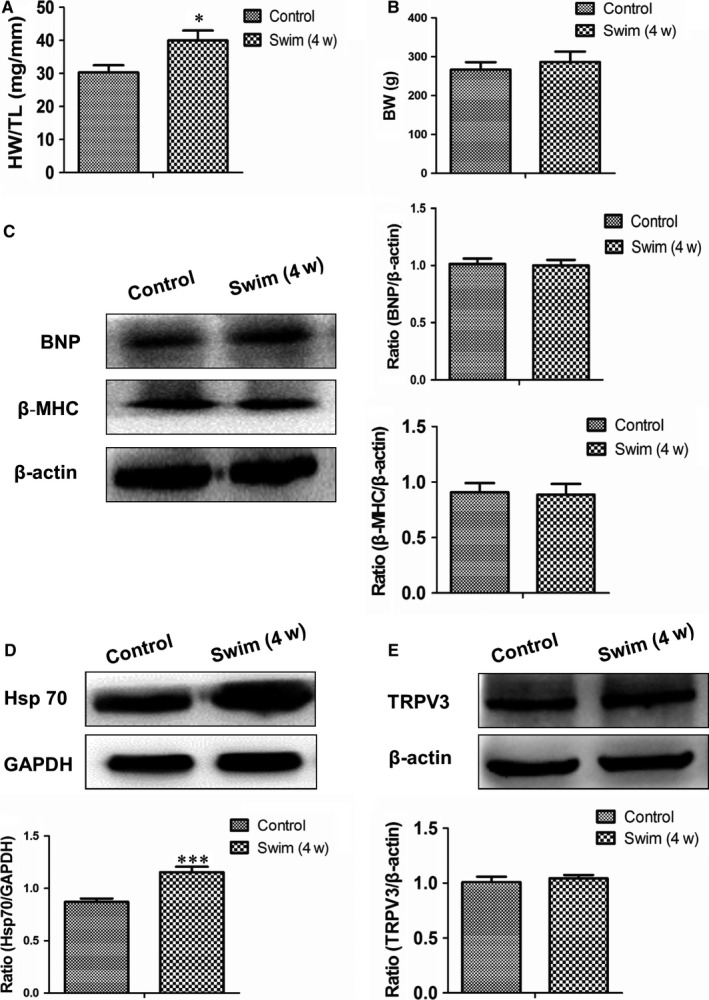
No change of TRPV3 expression in rat heart after 4 weeks of swimming exercise. A, The ratio of heart weight/tibial length (HW/TL) was increased after 4‐week swimming exercise in rat. B, Body weight after swimming exercise. C, BNP and β‐MHC proteins were not altered after 4‐week swimming exercise. D, Hsp70 protein was increased after 4‐wk swimming exercise. E, TRPV3 protein expression was not elevated after 4‐wk swimming exercise. *P < 0.05, ***P < 0.001 vs control group. Data were presented by mean ± SD (n = 6)

To identify the specificity of TRPV3 increase in pathological cardiac hypertrophy, we used the swimming training protocol to establish physiological cardiac hypertrophy. After 4 weeks swimming exercise, HW/TL was increased (*P *< 0.05) (Figure 2A), and body weight was no change in swim group compared with control group (Figure 2B). Pathological protein markers such as BNP and β‐MHC expressed also no alterations compared with control group (Figure 2C). It has been reported that expression level of the cardioprotective gene Hsp70 was increased in the exercise‐induced (physiological) hypertrophy but not in the pressure overload‐induced (pathological) hypertrophy model.^14^ Our results also confirmed that the expression of Hsp70 protein was increased in the swimming exercise model (*P *< 0.001) (Figure 2D) not in the AAC model (Figure 1D). Thus, we concluded that swimming exercise induced physiological cardiac hypertrophy. TRPV3 protein expressed no change in physiological hypertrophy (Figure 2E). The present work indicated that enhancive TRPV3 was specific in pathological cardiac hypertrophy, not in physiological cardiac hypertrophy.

### 3.2 Effects of TRPV3 on Ang II‐induced cardiacmyocytes hypertrophy in vitro

**Figure 3:**
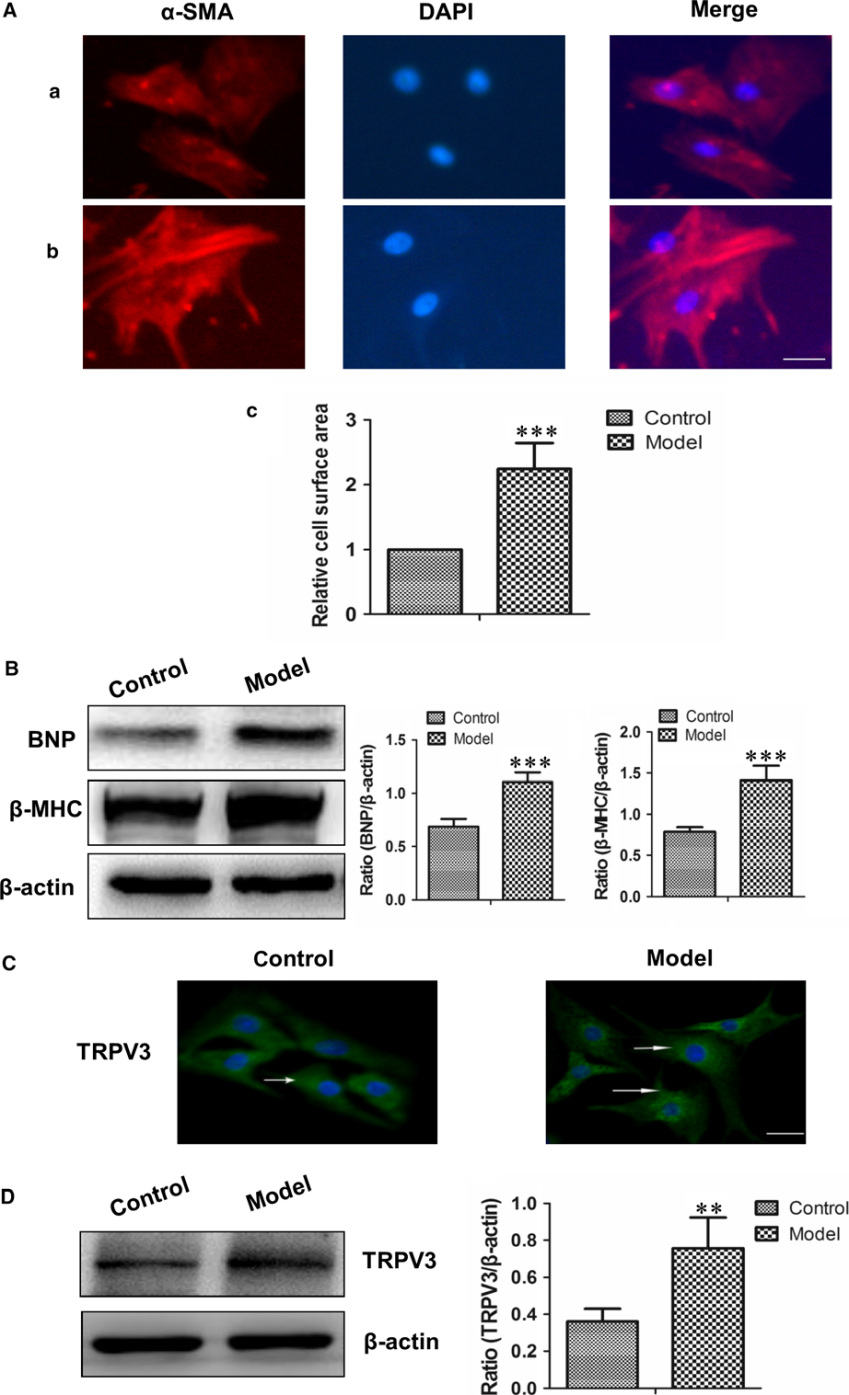
TRPV3 expression was increased in pathological cardiac hypertrophy induced by Ang II in vitro. A, Ang II induced significant increase in cell surface of cardiomyocytes. Cardiomyocytes were identified with sarcomeric α‐actinin antibody (red), and nuclei were stained with DAPI (blue) (×200). a, Control group; b, Model group: 100 nmol L^−1^ Ang II; c, Summarized data of cell area (n = 6). Scale bars are 25 μm. B, Ang II induced increases in BNP and β‐MHC proteins expression in cardiomyocytes (n = 4). C, Cardiomyocytes were fixed and stained with anti‐TRPV3 (green) and DAPI to stain nucleus (blue), (n = 6). Scale bars are 25 μm. D, TRPV3 protein expression was increased in cardiomyocytes treated with Ang II for 48 h in vitro (n = 4). **P < 0.01, ***P < 0.001 vs control group. Average data were represented by mean ± SD

To investigate TRPV3 expression in hypertrophy cardiomyocytes, we established a model of 100 nmol L^−1^ Ang II for 48 h. Ang II induced significant increases in cell surface area, BNP and β‐MHC (pathological markers) expressions of cardiomyocytes (Figure 3A, B), meanwhile, the expression of TRPV3 was also increased in the presence of Ang II (*P *< 0.01) (Figure 3C, D). The results suggested that TRPV3 expression was elevated in Ang II‐induced cardiacmyocytes hypertrophy.

**Figure 4:**
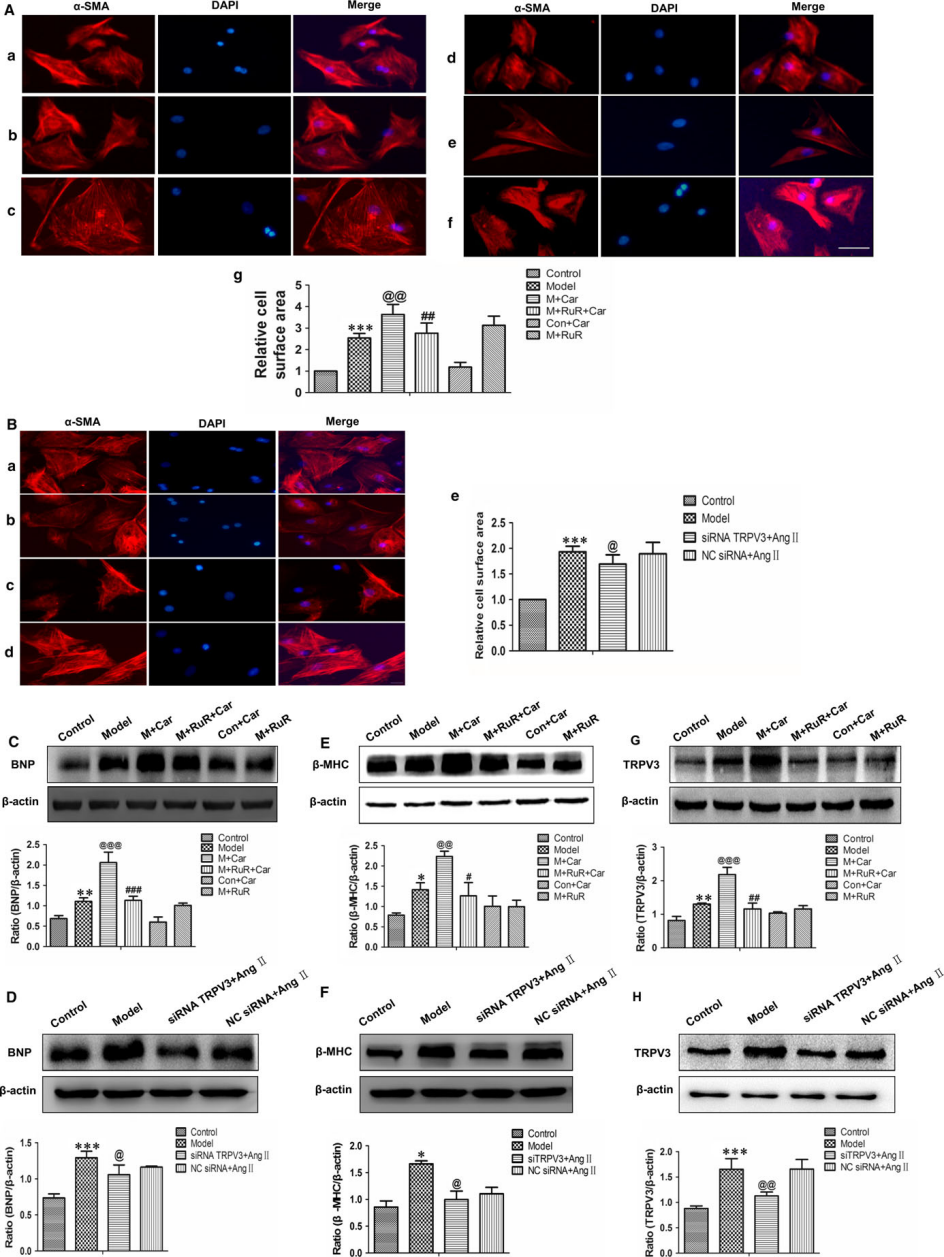
Cardiac hypertrophy was aggravated by the activation of TRPV3. A and B, Cardiomyocytes were identified with sarcomeric α‐actinin antibody (red), and nuclei were stained with DAPI (blue) (×200). A, (a) Control group; (b) Model group; (c) Model + carvacrol group; (d) Model + ruthenium red + carvacrol group; (e) Control + carvacrol group; (f) Model + ruthenium red group; (g) Summarized data of cell area. B, (a) Control group; (b) Model group; (c) siRNA TRPV3 + Ang II group; (d) NC siRNA + Ang II group; (e) Summarized data of cell area. C and D, BNP expression was analysed by western blot in cultured cardiomyocytes. E and F, β‐MHC expression was analysed by western blot in cultured cardiomyocytes. G and H: TRPV3 expression was analysed by western blot in cultured cardiomyocytes. *P < 0.05, **P < 0.01, ***P < 0.001 vs control group; ^@^ *P *< 0.05, ^@@^ *P *< 0.01, ^@@@^ *P *< 0.001 vs model group; ^#^ *P *< 0.05, ^##^ *P *< 0.01, ^###^ *P *< 0.001 vs model + carvacrol group. Average data were represented by mean ± SD (n = 4). Scale bars are 25 μm. Ang II: 100 nmol L^−1^; carvacrol: 60 μmol L^−1^; ruthenium red: 20 μmol L^−1^

Next, we further investigated the role of TRPV3 activation in cardiomyocyte hypertrophy. As showed in Figure 4A‐b, cardiac myocyte surface was significantly increased by 100 nmol L^−1^ Ang II (*P *< 0.001). Cardiomyocytes showed a dramatic increase in cell surface area after 4 hours exposure to 60 μmol L^−1^ carvacrol in Ang II‐induced hypertrophy for 48 hours (*P *< 0.01) (Figure 4A‐c). Moreover, pretreatment with 20 μmol L^−1^ ruthenium red, a nonselective TRPV channel antagonist, reduced the effect of carvacrol after treatment with 100 nmol L^−1^ Ang II (*P *< 0.01) (Figure 4A‐d). Meanwhile, cardiomyocytes were treated with carvacrol alone showing no effect compared with control group (Figure 4A‐e). The treatment of ruthenium red in Ang II‐induced cardiomyocytes showing a similar effect on Ang II model (Figure 4A‐f). Because of carvacrol and ruthenium red being nonselective TRPV channel agonist and antagonist, we next used specific siRNA TRPV3 technology to examine the effect of TRPV3 in cardiomyocytes. As shown in Figure 4B, when the TRPV3 was knocked down, cardiac myocyte surface was reduced compared with model group (*P* < 0.05) (Figure 4B‐e). Next, BNP, β‐MHC and TRPV3 protein expressions were measured by Western blot (Figure 4C–H). The changes of proteins appeared the same tendency as the changes of cell surface in each group. These results showed that the activation of TRPV3 aggravated cardiac hypertrophy.

### 3.3 Effects of TRPV3 on [Ca^2+^]_i_ in Ang II‐induced cardiacmyocytes hypertrophy

**Figure 5:**
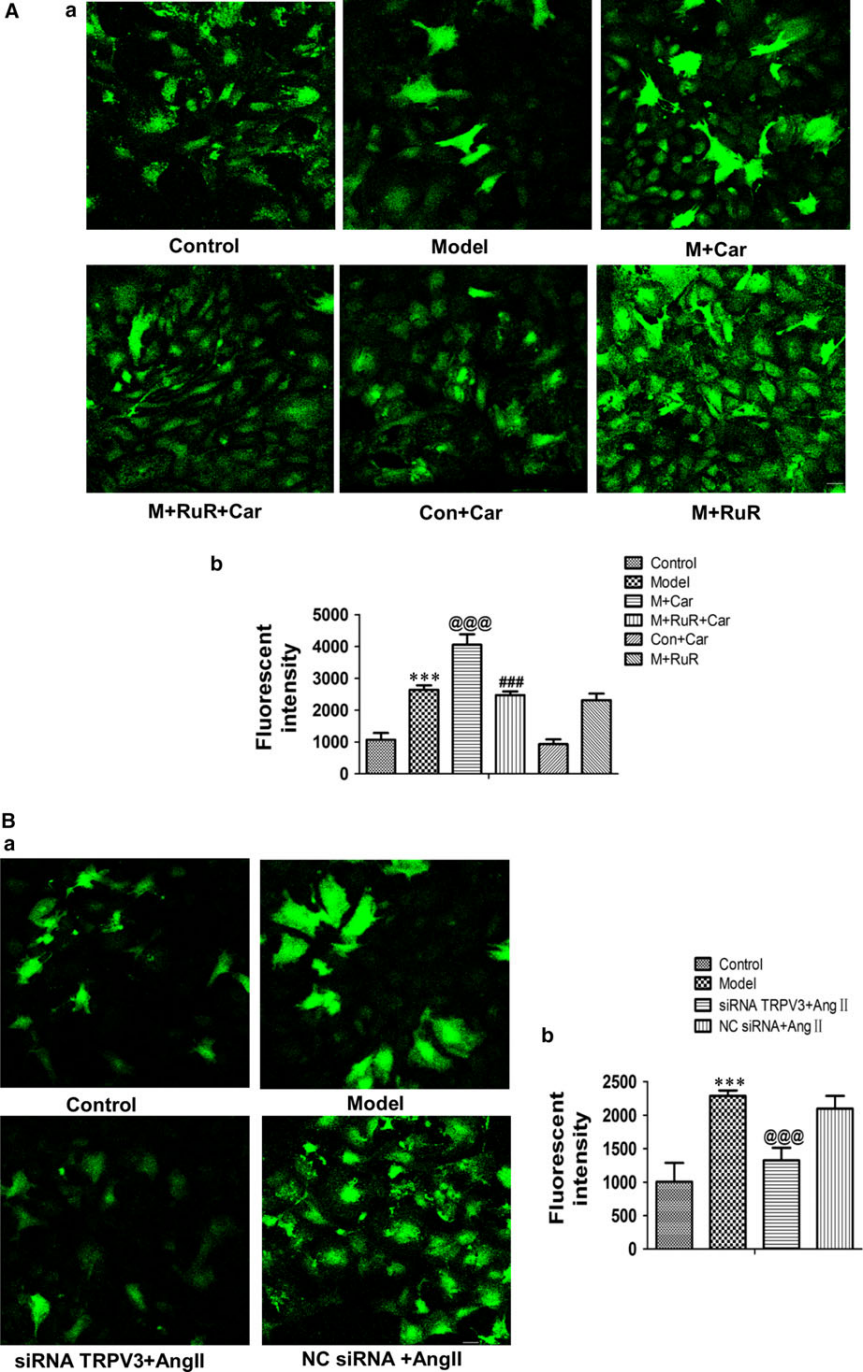
Ca^2+^ fluorescence measurement in cultured cardiomyocytes. Fluorescent intensity in intracellular calcium ions was recorded by laser scanning confocal microscope. Representative images of cardiomyocytes in different treatment (A‐a and B‐a). Quantitative analysis about fluorescence intensity (A‐b and B‐b). ***P < 0.001 vs control group; ^@@@^ *P *< 0.001 vs model group; ^###^ *P *< 0.001 vs model + carvacrol group. Data were presented by mean ± SD (n = 6). Groups in A were control, model, model + carvacrol, model + ruthenium red + carvacrol, control + carvacrol and model + ruthenium red. Groups in B were control, model, siRNA TRPV3 + Ang II and NC siRNA + Ang II. Ang II: 100 nmol L^−1^; carvacrol: 60 μmol L^−1^; ruthenium red: 20 μmol L^−1^

In our study, we investigated the effects of TRPV3 on the levels of intracellular calcium ions in Ang II‐induced cardiacmyocytes hypertrophy. The results showed that Ang II increased Ca^2+^ and combination stimulus of carvacrol remarkably induced increase in fluorescent intensity (*P *< 0.001) (Figure 5A‐a). The addition of ruthenium red for 2 hours before the carvacrol following Ang II stimulus attenuated Ca^2+^ compared with carvacrol stimulus (*P *< 0.001) (Figure 5A‐a). Moreover, normal cardiomyocytes and the addition of carvacrol showed similar low fluorescent intensity, Ang II condition and ruthenium red for 4 hours on Ang II showed similar high fluorescent intensity (Figure 5A‐b). When silencing the TRPV3, [Ca^2+^]_i_ was decreased compared with model group (*P *< 0.001) (Figure 5B). Together, these data suggested that TRPV3 activation could promote the increase in intracellular calcium concentration.

### 3.4 TRPV3 activation contributed to cardiac hypertrophy by regulating calcineurin/NFATc3 signalling pathway

**Figure 6:**
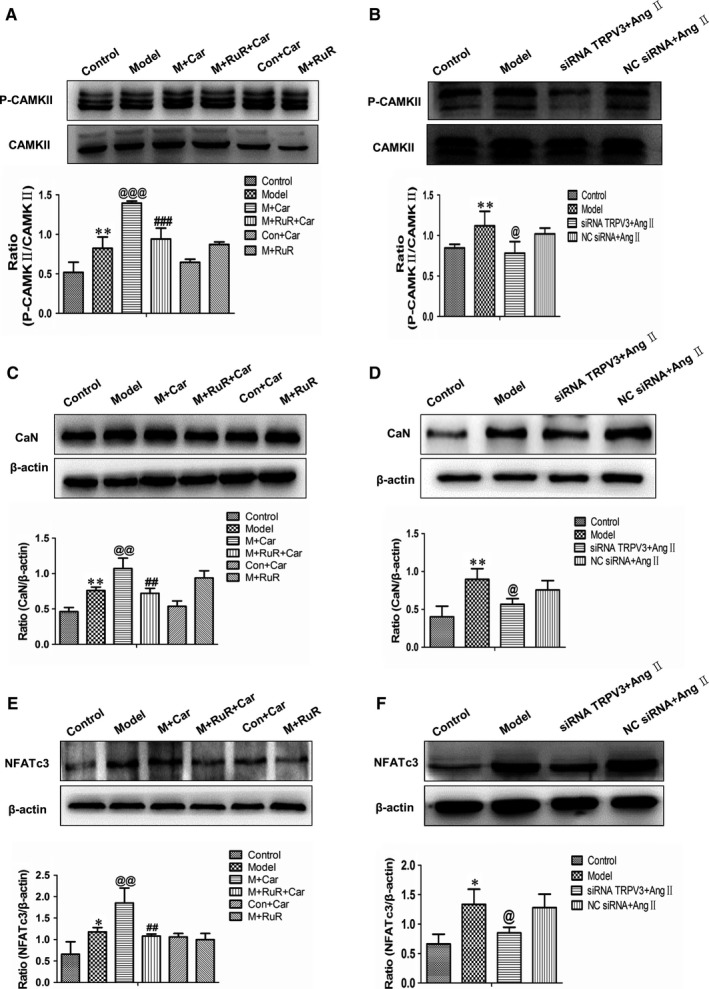
TRPV3 activation contributed to cardiac hypertrophy by regulating calcineurin/NFATc3 signalling pathway. P‐CAMKII (A and B), calcineurin (C and D) and NFATc3 (E and F) proteins expressions were analysed by western blotting. *P < 0.05, **P < 0.01 vs control group; ^@^ *P *< 0.05, ^@@^ *P *< 0.01, ^@@@^ *P *< 0.001 vs model group; ^##^ *P *< 0.01, ^###^ *P *< 0.001 vs model + carvacrol group. Data were shown as mean ± SD (n = 4). Ang II: 100 nmol L^−1^; carvacrol: 60 μmol L^−1^; ruthenium red: 20 μmol L^−1^

We have demonstrated that the activation of TRPV3 is related with the changes of Ca^2+^. To determine whether calcineurin/NFATc3 pathway was altered with TRPV3 changing, we investigated the levels of phosphorylated CaMKII protein. Western blot analyses indeed revealed remarkable incremental phosphorylated CaMKII protein in model group (*P *< 0.01), which was further enhanced in the presence of carvacrol (*P *< 0.001) and reduced by ruthenium red (*P *< 0.001). The phosphorylated CaMKII expression level was unchanged between control + carvacrol group and control group. In addition, this protein level in model + ruthenium red group as well as the model group was similar (Figure 6A). When TRPV3 was knocked down by siRNA technique, phosphorylated CaMKII expression was reduced compared with the model group (*P *< 0.05) (Figure 6B). Meanwhile, the protein expressions of calcineurin and NFATc3 (Figure 6C‐F) indicated the same trends as phosphorylated CaMKII.

Collectively, these results suggested that calcineurin/NFATc3 pathway contributed to the effects of TRPV3 on Ang II‐induced cardiacmyocytes hypertrophy.

## 4 DISCUSSION

**Figure 7:**
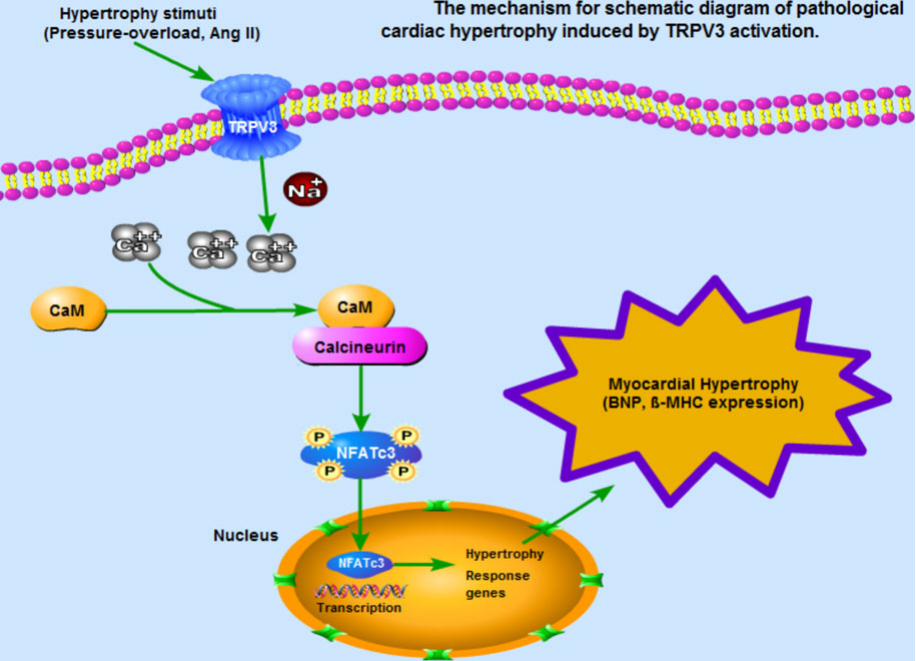
The mechanism for schematic diagram of pathological cardiac hypertrophy induced by TRPV3 activation. Under a variety of pathological stimuli, TRPV3 is activated, which in turn accelerates intracellular Ca^2+^ level in cardiac myocyte, further results in an increase in calcineurin/NFATc3 signalling pathway, and eventually leads to cardiac hypertrophy

In this study, to our knowledge, we demonstrated, for the first time, that the expression of TRPV3 was increased in pathological cardiac hypertrophy. The underlying mechanism of pathological cardiac hypertrophy induced by TRPV3 activation was summarized in Figure 7. We found that TRPV3 activation promoted cardiac hypertrophy via Ca^2+^/CaMKII/calcineurin/NFATc3 pathway.

In TRP subfamily, TRPV1 is known as a nonselective cationic channel and is also found in the heart and circulatory system.^15^ As an example, increasing TRPV1 channel expression was detected in hypertrophic myocardium.^15^ Importantly, TRPV3 was found to co‐express and form heteromultimers with TRPV1.^16^ In light of the close proximity between genes for TRPV1 and TRPV3, it is interesting to consider that TRPV3 is related with cardiac hypertrophy.

Previous investigations pay more attention to TRPV3 in the understanding of physiological mechanisms of nociception and thermosensation.^17^ Many lines of evidence from our studies supported that TRPV3 promoted cardiac hypertrophy via calcineurin/NFATc3 pathway. Firstly, a significant finding of this study was that the specific TRPV3 expression was increased in pathological cardiac hypertrophy induced by abdominal aortic constriction and Ang II stimulation under the increase in cardiac‐specific BNP and β‐MHC, not in swimming exercise model. Furthermore, we observed that the cell surface was remarkly increased by TRPV3 nonselective channel agonist carvacrol. Ruthenium red, TRPV3 nonselective channel antagonist, reversed the effects of carvacrol. In order to ascertain that TRPV3 expression in cardiomyocytes was active, we silenced TRPV3 and found that cell surface increased by Ang II was significantly reduced. This finding appeared to provide the evidence that cardiac hypertrophy was associated with the activation of TRPV3.

In cardiacmyocytes, intracellular calcium serves as an electrical signal and also induces hypertrophy or apoptosis genes. TRPV3 may play an important role in calcium influx and stimulate these activities. Based on TRPV3 being a member of the TRP family, we presumed that depletion of TRPV3 could induce changes of [Ca^2+^]_i_ in cardiomyocytes. In our results, we found that [Ca^2+^]_i_ was increased in Ang II‐treated neonatal rat cardiomyocytes and significantly enhanced in combination stimulus of carvacrol, and further attenuated by ruthenium red. When TRPV3 was knocked down by siRNA technology, [Ca^2+^]_i_ was remarkably decreased by laser scanning confocal microscope. This finding appeared to provide further evidence that TRPV3 activation indeed promoted the release of intracellular Ca^2+^.

In addition, calcineurin/NFATc3 hypertrophic signalling pathway plays a pivotal role in cardiac hypertrophy. A number of pathological stimuli can trigger the increment of intracellular calcium in cardiac myocytes. Increased Ca^2+^ binds to calmodulin (CaM) and activates the phosphates calcineurin and thereby induces nuclear translation of NFAT, which is contributed to the molecule signalling pathway in pathological cardiac hypertrophy. CaMKII is activated when Ca^2+^ is binding to CaM, which is also associated with pathological cardiac stress, mechanical dysfunction, arrhythmias, and myocyte apoptosis.^18^ Nilius et al revealed that a Ca^2+^‐CaM‐binding site was identified at the N‐terminus of the TRPV3.^19^ Also, to repeated stimulations, Ca^2+^ plays a central role in the sensitization of TRPV3.^20^ Our study showed that TRPV3 activation facilitated the phosphorylation of CaMKII, calcineurin, and NFATc3 expression and the increased effects in the presence of carvacrol were reduced by ruthenium red. When TRPV3 was silenced by siRNA technique, phosphorylated CaMKII, calcineurin, and NFATc3 expressions were reduced compared with model group. Taken together, our results supported this idea that calcineurin/NFATc3 pathway was involved in the cardiac hypertrophy induced by the activation of TRPV3.

In our study, agonist (carvacrol) and antagonist (ruthenium red) compounds were remarkably nonselective. Ruthenium red, for example, blocks both TRPV1 and TRPV3. While carvacrol activates TRPV3, it also has effects on TRPA1. Therefore, we should discuss whether TRPV1 and TRPA1 also contribute to cardiac hypertrophy. It has been reported that increasing TRPV1 channel expression was involved in cardiac hypertrophy. However, the effect of TRPA1 on cardiac hypertrophy was not clear. Therefore, we added further experiments to assess the effect of TRPA1 on cardiac hypertrophy by silencing TRPA1. We detected the protein expressions of β‐MHC, calcineurin and NFATc3 by Western blot analysis. The results were shown in Figure S1. When TRPA1 was silenced by siRNA technique, the expressions of β‐MHC, calcineurin and NFATc3 were reduced. So, we speculated that TRPA1 appeared similar effects with TRPV3. TRPA1 may aggravate the process of cardiac hypertrophy. In our experiment, we could not exclude the damage effect of TRPV1 and TRPA1 activation on cardiac hypertrophy when ruthenium red and carvacrol were applied in vivo or in vitro. Therefore, we used siRNA technology to silence TRPV3 specifically in vitro to explore the effect of TRPV3 on cardiomyocyte hypertrophy. Our results indicated that cardiac hypertrophy was alleviated by silencing TRPV3. Pires et al demonstrated that carvacrol increased the frequency of unitary Ca^2+^ influx events through TRPV3 channels in endothelial cells. And the amount of Ca^2+^ influx during the opening of a single TRPV3 channel is greater than a single TRPA1 channel.^21^ So, we thought that TRPV3 activation, at least partially, aggravated cardiac hypertrophy in our experimental condition.

As shown in Figure 7, our findings showed new insights about the role of TRPV3 channel in the regulation of pathological cardiac hypertrophy. In summary, our study established that TRPV3 was activated by hypertrophic stimulus, and intracellular calcium concentration was increased, which in turn activated calcineurin/NFATc3 pathway, and eventually led to cardiac hypertrophy.

## CONFLICT OF INTEREST

The authors confirm that there are no conflicts of interest.

## AUTHOR CONTRIBUTIONS

Conceived and designed the research study: Hongli Sun. Performed the research: Qianhui Zhang, Hanping Qi, Yonggang Cao, Pilong Shi, Chao Song, Lina Ba, Yunping Chen, Jingquan Gao, Shuzhi Li, Baiyan Li. Analysed the data: Qianhui Zhang, Hanping Qi. Wrote the paper: Qianhui Zhang, Baiyan Li, Hongli Sun.
