# Supplementary material for: Activation of transient receptor potential vanilloid 3 channel (TRPV3) aggravated pathological cardiac hypertrophy via calcineurin/NFATc3 pathway in rats
Source: J Cell Mol Med. 2018 Oct 9;22(12):6055–67. doi: 10.1111/jcmm.13880 (PMC6237578; doi:10.1111/jcmm.13880)

**
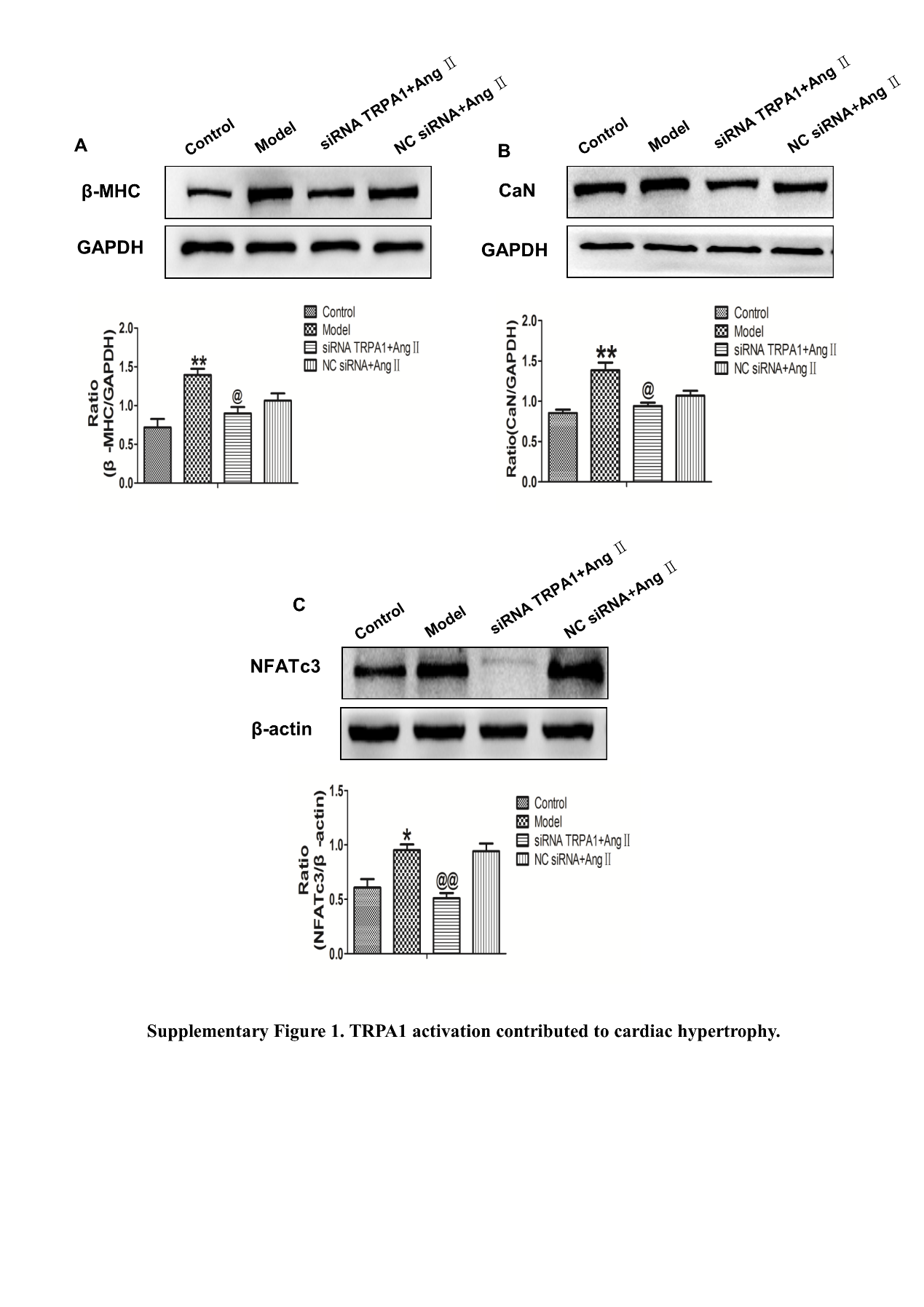
**

**Supplementary figure legends**

**Supplementary Figure 1. TRPA1 activation contributed to cardiac hypertrophy.** β-MHC (**A**), calcineurin (**B**) and NFATc3 (**C**) proteins expressions were analyzed by western blotting. ^*^*P* < 0.05, ^**^*P* < 0.01 *vs*. control group; ^@^*P* < 0.05, ^@@^*P* < 0.01 *vs.* model group. Data were shown as mean ± SD (n = 3). Ang II: 100 nM.


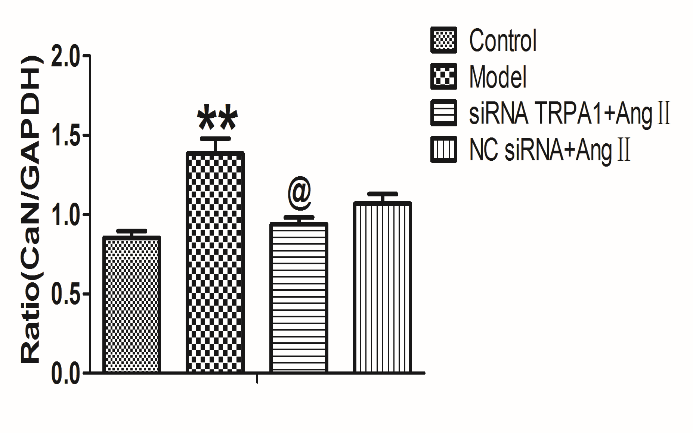

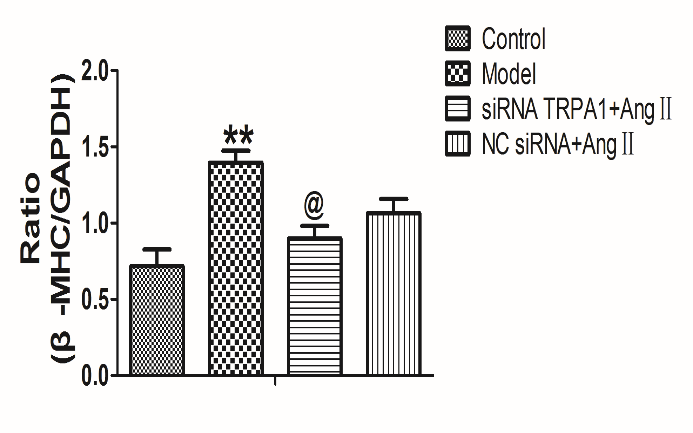

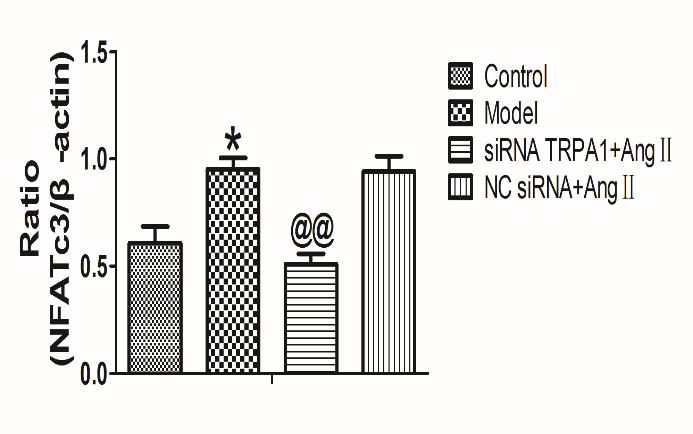


**β-actin**

**C**

**NFATc3**


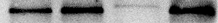

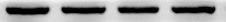


**B**

**GAPDH**

**CaN**


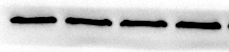

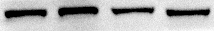

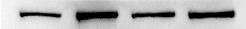


**GAPDH**

**β-MHC**

**A**


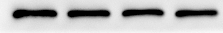

Supplement: Supplementary file 1 [file JCMM-22-6055-s001.docx]
